# Evolution of the Defense Compounds Against Biotic Stressors in the Invasive Plant Species *Leucaena leucocephala*

**DOI:** 10.3390/molecules30112453

**Published:** 2025-06-03

**Authors:** Hisashi Kato-Noguchi, Midori Kato

**Affiliations:** Department of Applied Biological Science, Faculty of Agriculture, Kagawa University, Miki 761-0795, Kagawa, Japan

**Keywords:** allelochemical, biotic stressor, herbivore, insecticide, invasive species, mimosine, nematicide, pathogen, pesticide

## Abstract

*Leucaena leucocephala* (Lam.) de Wit is listed in the world’s 100 worst alien invasive species because of the risks it poses to native plant communities. Life history traits, such as high growth and reproductive rates, and a high capacity to adapt to different environmental conditions may contribute to its invasive properties. Biotic stressors, such as herbivores, pathogens, and competing plant species are known to exert significant selective pressure on the plant’s survival, distribution, and abundance. *L. leucocephala* has been reported to contain several compounds involved in the defense functions against these biotic stressors. A large amount of L-mimosine, a non-protein amino acid, was found in all plant parts of *L. leucocephala*, including its flowers. L-Mimosine is toxic to herbivorous mammals and insects, parasitic nematodes, pathogenic fungi, and neighboring competing plant species by inactivating various essential enzymes and blocking DNA replication, and/or inducing oxidative stress conditions. Several flavonoids, polyphenolic compounds, and/or derivatives of benzoic and cinnamic acids are toxic to parasitic nematodes, pathogenic fungi and bacteria, and competing plant species by disrupting plasma membrane structures and functions, and various metabolic processes. These compounds may represent the invasive traits of *L. leucocephala* that have undergone natural selection during the evolution of the species. They may contribute to the defense functions against the biotic stressors, and increase its survival, distribution, and abundance in the introduced ranges. This is the first review to focus on the compounds involved in the defense functions against biotic stressors.

## 1. Introduction

**Figure 1:**
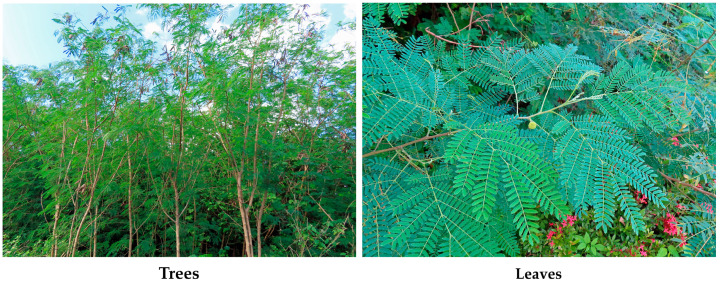
Trees and leaves of *L. leucocephala*.

*Leucaena leucocephala* (Lam.) de Wit, belonging to the Fabaceae family, is a perennial tree, 3–10 m, rarely 20 m, in height, with a cylindrical stem of 5–50 cm in diameter and branched. The leaves are alternate and bipinnate, consisting of 4–9 pairs of pinnae per leaf, and 13–21 pairs of leaflets per pinna [1,2,3,4]. The lifespan of *L. leucocephala* is over 30 years [5], and the annual biomass productivity has been estimated to be at 20–60 tons per hectare in the tropical regions [6] (Figure 1).

The native range of *L. leucocephala* is Mexico and Central America, and the species has been introduced worldwide for its economic benefits, such as fodder for livestock [7,8,9,10], shade trees in nurseries and plantations [11,12,13], timber, paper pulp and biochar production [14,15,16,17], and rehabilitation and erosion control to cover degraded lands and slopes [18,19,20]. However, *L. leucocephala* is an aggressive colonizer, easily escaping to unintended sites, and forming dense monospecific stands in riparian areas, grasslands, hillsides, forest edges, abandoned agricultural fields, disturbed areas, roadsides, and protected areas, including reserved forests [5,21,22]. The expansion rate of the *L. leucocephala* population on the main island of Taiwan was estimated to be 3.4 hectare per year [23]. *L. leucocephala* has already spread into more than 130 countries in the tropical, subtropical, and warm temperate regions of South America, Africa, South and East Asia, Southern Europe, Australia, and the islands in the Caribbean, Indian, and Pacific Oceans [2,5,24].

*L. leucocephala* threatens native plant communities, reducing the density and richness of the native plant species, including endemic and endangered plant species in the infested areas [5,21,25]. The establishment of the native tree species in areas infested by *L. leucocephala* was hardly observed [25,26,27,28]. Field experiments have confirmed that the presence of *L. leucocephala* suppressed the regeneration process, such as germination and growth, of the native woody plant species *Erythrina velutina* Willd. [25]. The plant communities in the islands are much more vulnerable to the infestation of *L. leucocephala*, and the native woody species, including protected plant species, were replaced by *L. leucocephala* due to the interruption of their regeneration processes [2,26,27,28,29,30,31,32,33,34,35]. The infestation of *L. leucocephala* has also altered the plant successional pathway, and reduced the plant diversity in the mid- and late-successional forests. The dominance of *L. leucocephala* has persisted for a long time, and the composition of the native forests have not recovered [26,34,35].

Soil nitrogen levels under the *L. leucocephala* stands were high due to the symbiotic nitrogen fixation [36,37,38]. The increase in soil nitrogen availability may affect the composition of the existing plant species, and the structure and function of the ecosystem. As a result, the food webs and invertebrate and vertebrate habitats may be altered [39]. Because of the risk of invasion into native plant communities, *L. leucocephala* has been listed in the world’s 100 worst alien invasive species by the International Union for Conservation of Nature and Natural Resources [40]. The global warming trend may further increase the potential risk of *L. leucocephala* invasion [41].

**Figure 2:**
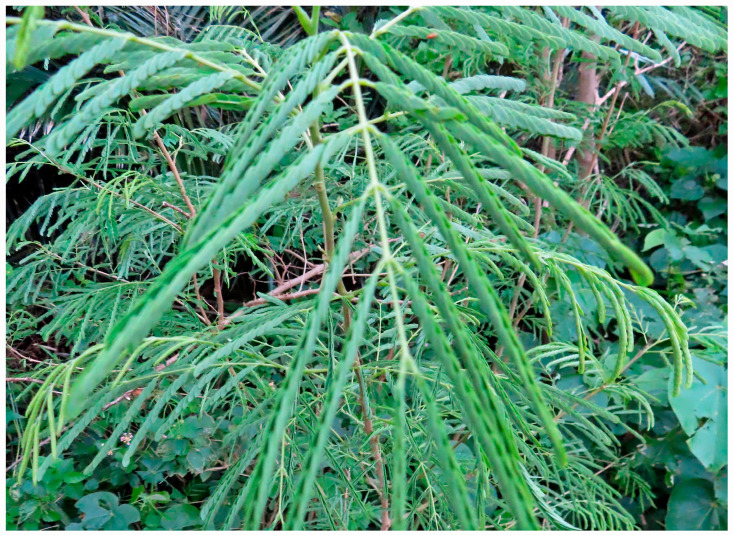
Closure of the leaves of *L. leucocephala* during daytime.

To understand the invasion mechanism, the life history traits of several invasive plant species, such as their ability to adapt to different environmental conditions and their growth and reproductive capabilities, have been documented [42,43,44,45,46,47]. This information may also be necessary to understand the invasion mechanism of *L. leucocephala. L. leucocephala* grows well in tropical and subtropical climates, between 15 and 25 degrees of north and south latitude of the globe, with annual precipitation ranging from 650 mm to 3000 mm, and temperatures from 18 to 30 °C, and on drained, neutral to slightly alkaline soils [2,4]. However, the species tolerates annual precipitation as low as 300 mm, and at 0 °C for the mean temperature of the coldest month, and up to 40 degrees of north and south of the equator, including a warm temperate climate [2,5]. *L. leucocephala* has drought tolerance through a strong ability to maintain leaf water content by reducing the transpiration without defoliation. The species can survive for several months under drought stress conditions, and can recover soon after receiving the available water. Its leaflets hold together with the opposite leaflets of the pinnae to avoid water transpiration from the leaves under drought stress conditions during the daytime, and the leaves also show nyctinasty to avoid transpiration during the nighttime [48,49,50]. This tolerance allows the species to adapt to different environmental conditions (Figure 2).

**Figure 3:**
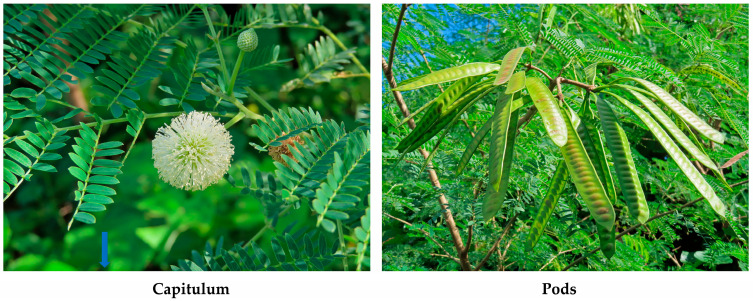
Capitulum and pods of *L. leucocephala*.

*L. leucocephala* grows rapidly and reaches its reproductive stages within 4–12 months after germination. The species flowers and produces seeds mainly by self-fertilization throughout the year under suitable climatic conditions [51,52,53]. The flat fruit pods contain 8–30 (average 18) seeds per pod [53,54]. The annual production rate of pods was recorded as 277–388 per plant, containing 4000–6000 seeds in tropical coastal Australia [53]. The species also dropped 5500 seeds per m^2^ per year on the forest floor in Brazil [55]. Seeds are carried by wind, water flow, and the activities of birds, rodents, and humans. Some seeds were carried by wind over 100 m from the parent plants [2,53]. Germination occurs over a long period of time after seed dispersal. The germination rate of fresh seeds was 59% under optimal conditions, and the seeds retained the ability to germinate for 20 years under good seed bank conditions [5,55,56]. In light of these observations, the life history traits, such as year-round flowering and fruiting, self-fertility, prolific seed production, seed bank establishment, and adaptability to different environmental conditions, may contribute to the infestation and expansion of *L. leucocephala* populations in new habitats (Figure 3).

*L. leucocephala* has also been reported to exhibit toxic activity against herbivorous mammals and insects, parasitic nematodes, pathogenic fungi and bacteria, and neighboring competing plant species, and to contain the compounds responsible for these toxicities. These compounds may be involved in the defense functions against biotic stressors, such as herbivores, pathogens, and competing plant species. Such defense functions may contribute to the successful infestation, naturalization, and expansion of invasive plants in the introduced range [57,58,59,60,61,62]. However, no review has focused on the toxicity and related compounds of *L. leucocephala* for the understanding of its invasive properties. This is the first review to provide an overview of the toxicity of *L. leucocephala* to biotic stressors and the specific compounds associated with the toxicity. The possible mechanisms of action of these compounds, and their involvement in the invasive characteristics of *L. leucocephala* were also discussed. The literature was searched using a combination of major online search engines, including Scopus, Google Scholar, and ScienceDirect, and the combinations of *L. leucocephala* with the following keywords: ecology, distribution, invasion, impact, habitat, adaptation, growth, reproduction, herbivore, mammal, pathogen, nematicide, insecticide, fungicide, pesticide, and allelopathy.

## 2. Toxicity Against Herbivorous Mammals

**Figure 4:**
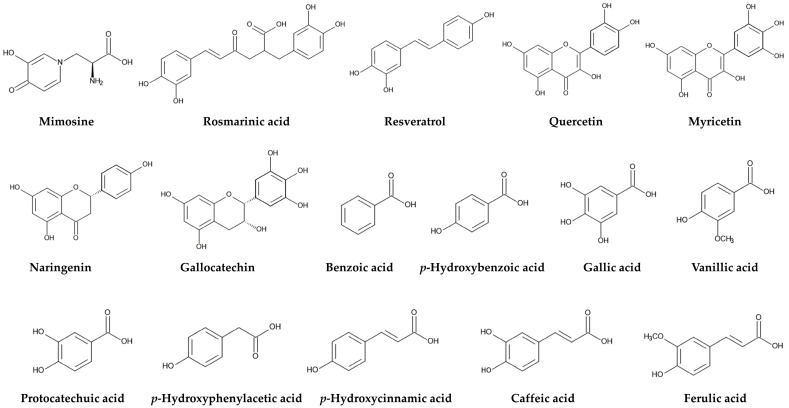
Compounds involved in defense functions of *L. leucocephala* against herbivorous mammals and insects, parasitic nematodes, pathogenic fungi and bacteria, and competing plant species. Polyphenolic compounds: rosmarinic acid, resveratrol. Flavonoids: quercetin, myricetin, naringenin, gallocatechin. Benzoic acid and cinnamic acid derivatives: benzoic acid, *p*-hydroxybenzoic acid, gallic acid, vanillic acid, protocatechuic acid, *p*-hydroxyphenylacetic acid, *p*-hydroxycinnamic acid, caffeic acid, ferulic acid.

*L. leucocephala* poisoning has been reported in ruminants, such as cattle (*Bos taurus* L.) [63,64,65], goats (*Capra hircus* L.) [66,67], and sheep (*Ovis aries* L.) [68], and in nonruminants, such as horses (*Equus caballus* L.) [69], pigs (*Sus scrofa domesticus* Erxleben) [70], rabbits (*Oryctolagus cuniculus* L.) [71,72], rats (*Rattus norvegicus* Berkenhout) [73,74,75], mice (*Mus musculus* L.) [76], and ring-tailed lemurs (*Lemur catta* L.) [77]. An active component of *L. leucocephala* poisoning has been identified as a non-protein amino acid, L-mimosine (hereafter, mimosine). Mimosine has been found in several species of the genera *Mimosa* and *Leucaena* [78,79,80] (Figure 4).

**Figure 5:**
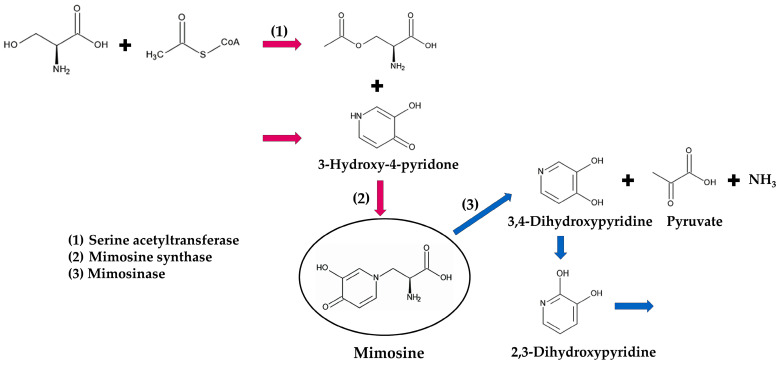
Mimosine biosynthesis and degradation pathway. Red arrows: biosynthesis; blue arrows: degradation.

*L. leucocephala* contains a large amount of mimosine in all parts of the plant. The concentration of mimosine was 2.4–13.6% (mimosine weight/dry weight of the plant) in mature seeds, 0.47–8.6% in leaves, 1.2–2.7% in flowers, 0.15–0.68% in stems, and 0.16–0.66% in roots [81,82,83]. It was 22.2% in young shoot tips, 14.7% in mature shoot tips, 6.4% in young leaves, and 1.4% in senescent leaves, indicating that newly developing tissues contain more mimosine than old tissues. Mimosine concentration was also increased during germination and seedling growth. The concentration was 3.9% in seeds, and increased to 6.9% at 6 days after the sowing, and was 15.2% in 2-week-old seedlings [82,83], indicating that most of the mimosine is synthesized de novo during germination and development. Mimosine is synthesized by mimosine synthase from *O*-acetyl-L-serine and 3-hydroxy-4-pyridone (3H4P) [84,85]. *O*-acetyl-L-serine is formed from serine and acetyl-CoA by serine acetyltransferase [84,85,86,87]. However, the pathway of 3-hydroxy-4-pryridone synthesis has not yet been confirmed in *L. leucocephala* (Figure 5).

Ingestion of the *L. leucocephala* leaves and seeds, and the administration of mimosine has caused decreases in the food intake, body weight gain, and leukocytes, and increases in alopecia, cataracts, hepatic congestion, and infant mortality in ruminants and nonruminants [64,65,66,67,68,69,70,71,72,77]. *L. leucocephala* and mimosine has also caused increases in infertility, abortion, and abnormal embryo development in female mammals, and decreases in the serum testosterone levels and sexual behavior in male mammals [73,75,76,88,89].

Goiter is also a major symptom of feeding on *L. leucocephala* and mimosine, which has been well documented in ruminants [90,91,92,93]. Rumen bacteria, such as *Synergistes jonesii* Allison, *Butyrivibrio fibrisolvens* Bryant & Small, and *Clostridium butyricum* Prazmowski, produce mimosinase, which metabolizes mimosine to 3,4-dihydroxypyridine (3,4-DHP), pyruvate, and ammonia, and certain rumen bacteria, possibly including *S*. *jonesii*, then convert 3,4-DHP to its isomer 2,3-dihydroxypyridine (2,3-DHP) [94,95,96,97]. The 3,4-DHP and 2,3-DHP are still toxic and cause goiter in ruminants [90,96,98]. Additional rumen bacteria may be involved in the further metabolism of 2,3-DHP to unidentified non-toxic substances [97,98,99,100]. The ingestion of *L. leucocephala* and mimosine has also caused the enlargement of the thyroid gland in rabbits, and abnormal thyroid cells, and/or decreased thyroid hormone levels in rats, rabbits, and horses [69,100,101,102,103], the mimosine metabolism has not yet been documented in nonruminants (Figure 5).

Some of the toxic effects of mimosine are thought to be caused by its chelating potential due to its pyridine ring and α-amino acid residue. Mimosine is capable of forming stable complexes with bivalent transition metal ions, such as Fe (II), Cu (II), Ni (II), and Zn (II) [98,104]. Mimosine inactivates several metal ion-dependent enzymes, such as alkaline phosphatase, ribonucleotide reductase, tyrosinase, and dopamine β-hydroxylase, by limiting the availability of the metal ions to the metabolic cofactors of these enzymes by chelating these ions [94,105,106]. Mimosine is also able to form a strong complex with pyridoxal-5′-phosphate (PLP), and inactivates PLP-dependent enzymes, such as cystathionine γ-lyase, aspartate aminotransferase, cysteine synthase, tyrosine decarboxylase, and L-DOPA decarboxylase [107,108,109,110]. Therefore, mimosine is able to interrupt several important metabolic processes by inhibiting the activity of the related enzymes.

Mimosine also inhibits the process of cell proliferation, specifically the transition from the G_1_ to the S phase. Mimosine is an effective cell cycle arresting reagent. It works by synchronizing cells within the late G1 phase by blocking entry into the S phase through the activation of the Ataxia-telangiectasia-mutated (ATM) gene, and interrupting the binding of the chromatin-associated protein Ctf4 to chromatin. It also prevents the formation of replication forks in the S phase, thereby blocking DNA replication [111,112,113]. As previously mentioned, mimosine inhibits ribonucleotide reductase activity [106]. Ribonucleotide reductase catalyzes the formation of deoxyribonucleotides from ribonucleotides, and regulates the elongation process of DNA replication in the S phase [114,115].

Considering these findings, it can be concluded that mimosine inactivates several essential enzymes and blocks the DNA replication process. Inactivation of the essential enzymes and inhibition of DNA replication may result in a variety of pathological symptoms, including alopecia, cataracts, hepatic congestion, goiter, increased infertility, abortion, abnormal embryo development in female mammals, and decreased serum testosterone levels in male mammals [64,65,66,67,68,69,70,71,72,73,74,75,76,77,88,89]. *L. leucocephala* contains a significant amount of the toxic substance mimosine in all parts of the plant. Therefore, mimosine may function to protect *L. leucocephala* from herbivory by herbivorous mammals (Figure 5).

## 3. Toxicity Against Herbivorous Insects

Herbivorous insects often cause significant limitations to the host plants, suppressing the growth, seed production, and survival [116,117,118,119]. Aqueous extracts of *L. leucocephala* leaves increased the mortality of the eggs and larvae of the silverleaf whitefly *Bemisia tabaci* Gennadius [120]. Hexane and dichloromethane extracts of *L. leucocephala* seeds increased the mortality of adults of the plant-feeding mite *Tetranychus urticae* C.L. Koch, and decreased their oviposition activity [121].

Mimosine inhibited the growth and development of the larvae of the Australian bollworm *Heliothis punctiger* Wallengren [122] and the red flour beetle *Tribolium castaneum* Herbst [123], and increased the mortality of the adult termite *Coptotermes formosanus* Shiraki with an LD_50_ value of 1.2 μM [124]. Mimosine also inhibited the activity of acetylcholinesterase and tyrosinase in the adult termite *C. formosanus*. The concentration of mimosine, required for 50% inhibition of these activities, was determined to be 31.4 and 46.1 μM for acetylcholinesterase and tyrosinase, respectively [124]. Acetylcholinesterase catalyzes the breakdown of acetylcholine, a neurotransmitter, to acetate and choline at the synapses in the nervous system [125,126]. Suppression of acetylcholinesterase results in the prolonged retention of this neurotransmitter at the synapses of the acetylcholine receptors. This prolonged retention leads to the sustained excitation of neurons, resulting in abnormal behavior, including death [126,127,128]. Tyrosinase is a copper-containing enzyme involved in tyrosine metabolism, which is responsible for cuticle hardening and immune responses in insects [129,130,131]. Therefore, the inhibition of acetylcholinesterase and tyrosinase by mimosine may inhibit the growth and development of insects, and increase their mortality. Mimosine has also been reported to arrest cell division between the G_1_ and S phases of the mosquito *Aedes albopictus* [132]. The inhibition of cell division, and the activity of acetylcholinesterase and tyrosinase by mimosine in these insects may be caused by a mechanical action similar to that described in the mammalian cells. The extracts of the seeds and leaves of *L. leucocephala* increased the mortality of the insects, and mimosine may be an active principal of the extracts. Therefore, mimosine may function to protect *L. leucocephala* from herbivory by herbivorous insects.

## 4. Toxicity Against Parasitic Nematodes

The root-knot nematodes *Meloidogyne* spp. are parasitic on various host plant species. The juveniles of *Meloidogyne* spp. invade plant root cells, and form permanent feeding sites and root-knot galls, known as root-knot disease. The infested nematodes absorb nutrients from the host plant through the feeding sites. The feeding process of the nematodes causes severe disease, such as yellowing, wilting and/or stunting. The feeding process also causes significant damages to the host plant’s root system, reducing its ability to absorb water and nutrients from the soil and to resist infection by other pathogens [133,134,135,136,137].

Aqueous, methanol, and ethanol extracts of the leaves, roots, stems, and seeds of *L. leucocephala* increased the mortality of the root-knot nematode *Meloidogyne incognita* Kofoid & White and decreased its egg hatching, population, and parasitism [138,139,140]. Aqueous extracts of *L. leucocephala* leaves also increased the mortality of another root-knot nematode *Meloidogyne exigua* Goeldi, and decreased its parasitism and oviposition activity [141]. Aqueous ethanol extracts of *L. leucocephala* seeds increased the mortality of the laboratory model nematode *Caenorhabditis elegans* Maupas with an LC_50_ value of 73 mg of the plants/mL, and decreased its movement, feeding, and oviposition activity [142]. Mimosine also increased the mortality of *C. elegans* with an LC_50_ value of 16.8 μM [124], and decreased its movement, feeding, and oviposition activity [142]. Quercetin (flavonoid) isolated from the leaves of *L. leucocephala* suppressed the eggs hatching of *M. incognita* and increased their mortality [143] (Figure 4). Quercetin also showed inhibitory activity on the eggs hatching of *Cooperia* spp., which is the common intestinal parasitic nematode of cattle [144], and increased the mortality of the larvae of the dog parasitic roundworm *Toxocara canis* Johnston [145]. Based on these findings, *L. leucocephala* has nematocidal activity and protects against nematode parasitism.

Given its effectiveness against mammals and insects, mimosine in *L. leucocephala* may contribute to the nematocidal activity of the extracts by inactivating enzymes and/or inhibiting cell proliferation. However, no direct research has been conducted on its effects on parasitic nematodes. The mode of action of quercetin on nematocidal activity has also not yet been reported.

## 5. Toxicity Against Pathogenic Fungi and Bacteria

Pathogenic microbes cause various significant diseases on the infested host plants, reducing their growth, biomass, reproduction, and survival [146,147,148]. Aqueous extracts of *L. leucocephala* leaves suppressed the spore germination and growth of the pathogenic fungi *Fusarium solani* (Mart.) Sacc., *Alternaria solani* Sorauer, and *Colletotrichum circinans* (Berkeley) Voglino [149]. Aqueous extracts of *L. leucocephala* seeds inhibited the growth of the pathogenic fungus *Sclerotium rolfsii* Saccardo. The extracts and mimosine caused the reduction of the mycelial protein and nucleic acid levels in *S. rolfsii* [150].

Aqueous methanol extracts of the seeds, leaves, stems, and roots of *L. leucocephala* also inhibited the growth of the pathogenic fungi *Rhizoctonia solani* J.G. Kühn, *F. solani*, and *A. solani*, as well as the pathogenic bacteria *Agrobacterium tumefaciens* (Smith & Townsend) Conn, and *Erwinia amylovora* Winslow. The main constituents of these extracts were as follows: rosmarinic acid (4.8 mg/g of dried plant part), resveratrol (3.0 mg/g), quercetin (2.1 mg/g), myricetin (1.4 mg/g), and naringenin (1.2 mg/g) in the leaf extracts; benzoic acid (0.6 mg/g) and naringenin (0.4 mg/g) in the root extracts; rosmarinic acid (2.2 mg/g), resveratrol (1.6 mg/g), and benzoic acid (1.1 mg/g) in the twig, stem, and bark extracts; and benzoic acid (1.5 mg/g), myricetin (0.8 mg/g), and rosmarinic acid (0.8 mg/g) in the fruit and seed extracts [151] (Figure 4). The mechanisms of the antifungal and antibacterial activity of flavonoids, such as quercetin, naringenin, and myricetin, have been reported to be the disruption of plasma membrane structures, resulting in the interruption of several physiological functions, including ATP synthesis [152,153]. The polyphenolic compound, resveratrol acts as a phytoalexin and inhibits the growth of various bacteria and fungi. Resveratrol interacts with more than 20 proteins, including ATP synthase in these microbes, resulting in the inhibition of ATP synthesis, induction of DNA fragmentation, and disruption of the membrane structure [154,155,156]. The other polyphenolic compound, rosmarinic acid has been reported to disrupt mitochondrial activity and cell membrane structures [157]. A possible mechanism of the antimicrobial activity of benzoic acid is considered to be hyper-acidification at the plasma membrane of the microbes due to the transport of its undissociated form across the cytoplasmic membrane into the cells, and the release of H^+^ ions. Hyper-acidification alters the functions of the cell membrane, making it more permeable, and disrupting the ATPase pump, leading to cell death [158,159,160]. Therefore, *L. leucocephala* has antifungal and antibacterial activity. Mimosine, benzoic acid, flavonoids, such as quercetin, naringenin, and myricetin, and polyphenolic compounds, such as rosmarinic acid and resveratrol, may be involved in these activities.

## 6. Toxicity Against Competing Plant Species

Plants fight with neighboring plants for niches and resources, such as light, nutrients, and water. Stronger competitive ability of the plants guarantees better conditions for survival [161,162,163,164,165,166,167]. Allelopathy is the phenomenon whereby the germination and growth of neighboring plants is affected by the release of allelochemicals. Several invasive plants have been reported to suppress the germination, growth, and fitness of neighboring competing plant species by releasing allelochemicals, resulting in the acquisition of many more resources [168,169,170,171,172]. Allelochemicals are produced and reserved in the host plant tissues, and released into the environment, including rhizosphere soils, as necessary through the volatilization, secretion, and degradation processes of plant residues in the soil [173,174,175,176,177]. The growth and survival of a woody plant species, *Erythrina velutina* Willd, was observed to be limited by the presence of *L. leucocephala* under relatively controlled field conditions [178]. The competition, between both species, for light, water, and nutrients was elucidated from the observations due to the relatively controlled experimental conditions, suggesting the possible involvement of the allelopathy of *L. leucocephala* in limiting the growth and survival of *E. velutina*. The allelopathic activity of *L. leucocephala* was determined in its extracts, plant residues, and rhizosphere soils. Several allelochemicals have also been identified.

Aqueous extracts of the whole aerial parts of *L. leucocephala* suppressed the growth of *Amaranthus hybridus* L. and *Bidens pilosa* L. under laboratory and greenhouse conditions [179], and aqueous extracts of the leaves and seeds of *L. leucocephala* inhibited the germination and growth of *Tridax procumbens* L., *Ageratum conyzoides* L., *Emilia sonchifolia* (L.) DC. ex Wight, *Pterogyne nitens* Tul., and *Peltophorum dubium* (Spreng.) Taub. [180,181]. Thus, the extracts of the aerial parts and seeds of *L. leucocephala* may contain certain allelochemicals that cause growth inhibition.

When the seeds of a woody plant, *Albiza procera* (Roxb.) Benth., were sown into a mixture of the soil and leaves of *L. leucocephala*, their germination and growth were suppressed [182]. Soil mixed with the decomposing leaves of *L. leucocephala* caused a decrease in survival of five woody plant species, including *Mimosa pudica* L., *Liquidambar formosana* Hance, *Casuarina glauca* Sieber ex Spreng., *Alnus formosana* (Burkill) Makino, and *Acacia confusa* Marr. [183]. Aqueous extracts of the litter accumulated on the soil beneath *L. leucocephala* plants suppressed the germination and growth of four herbaceous plant species, namely *Lolium multiflorum* Lam., *E. sonchifolia*, *T. procumbens*, and *A. conyzoides* [183,184]. Soil collected from under *L. leucocephala* stands suppressed the germination and growth of *E. sonchifolia*, *T. procumbens*, and *A. conyzoides*. Exudates from the roots of *L. leucocephala* also suppressed the germination and growth of these plant species [184]. Therefore, certain allelochemicals may be released into the rhizosphere soil through the root exudation and the decomposition process of plant litter and residues, causing the growth inhibition of these plant species.

Mimosine has also been identified as an allelochemical in *L. leucocephala* for its growth inhibitory activity against several plant species [183,184,185,186]. At a concentration of 100 ppm, mimosine inhibited the root growth of *B. pilosa*, *M. pudica*, *Phaseolus vulgaris* L., *Brassica rapa* L., and *L. multiflorum* by 40–95%, and their shoot growth by 31–93% [81]. Mimosine also inhibited the root and shoot growth of *Senna obtusifolia* (L.) H.S. Irwin & Barneby and *Sesbania herbacea* (Mill.) McVaugh [187], as well as *A. conyzoides*, *T. procumbens*, and *E. sonchifolia* [188]. Mimosine reduced the vigor of the *A. conyzoides* seedlings and wilted the seedling 7 days after application at 50 ppm [183].

On the other hand, mimosine suppressed the growth of the roots and shoots of *L. leucocephala* by only 2.4% and 12%, respectively, at a concentration of 100 ppm [81]. Thus, mimosine has relatively little inhibitory effect on *L. leucocephala* itself. The enzyme, mimosinase (Figure 5), which is responsible for the degradation of mimosine, was found in *L. leucocephala*, and the gene expression of the enzyme was high [189,190,191,192]. Therefore, mimosinase degrades mimosine, and the degradation process may attenuate the toxicity of the mimosine to *L. leucocephala* itself.

*L. leucocephala* seedlings released mimosine into the growth medium at 1–5 μg/g dry weight of the plant per day [193], and mimosine accumulated at 7.4 μg/g dry weight of the rhizosphere soil [194], suggesting that *L. leucocephala* may release mimosine into its rhizosphere soil. In addition, *L. leucocephala* contains a large amount of mimosine in whole plant parts. Some of the mimosine may also be released into the soil during the decomposition process of the plant residues, and accumulate in the soil under *L. leucocephala* trees. The accumulated mimosine in the soil may act as an allelochemical to suppress the germination and growth of neighboring plant species.

Aqueous extracts of the aerial parts of *L. leucocephala* inhibited cell division in the roots of *Zea mays* L. and *Pisum sativum* L. [195,196]. Mimosine also suppressed the cell division between the G_1_ and S phases of the protoplasts of *Petunia hybrida* Juss. [197] and the phytoplankton *Rhodomonas salina* Karsten [198]. Mimosine suppressed rooting by disrupting the root cell division in *Allium cepa* L. [199]. Therefore, mimosine may also disrupt the cell division process in plants, resulting in growth suppression.

Aqueous extracts of the aerial parts of *L. leucocephala* increased the peroxidase activity in the roots of *Z. mays* [179]. Rinsed water from *L. leucocephala* leaves increased the activity of ascorbate peroxidase and catalase in the leaves of *Eichhornia crassipes* (Martius) Solms-Laubach, and induced electrolyte leakage from their leaf cell membranes [200]. These enzymes are known to increase in plants under oxidative stress conditions [201,202,203]. Oxidative stress induces the alteration of the physiological processes and structures in plant cells, including the plasma membrane [204,205,206,207,208]. Mimosine also induced the abnormal cytoplasmic organelles and necrosis in the meristem cells of *A. cepa* root tips, and increased catalase and superoxide dismutase activities, and malondialdehyde levels in the roots [199,200,201,202,203]. Therefore, mimosine may induce oxidative stress conditions in the target plant species, and disrupt their physiological processes and cell structures.

Jasmonic acid, and ethephon which is metabolized into ethylene in plants after absorption, increased mimosine production and concentrations in *L. leucocephala* seedlings [209]. Salicylic acid, mechanical injury, UV irradiation, and NaCl treatment also increased the mimosine concentrations in the seedlings [82,209,210]. Salicylic acid, jasmonic acid, and ethylene are plant hormones that act as stress signaling molecules, and induce the expression of stress-related genes [211,212,213]. The stress conditions caused by the mechanical injury, UV irradiation, and NaCl treatment, as well as the stress signaling molecules, including salicylic acid, jasmonic acid, and ethylene, may stimulate the production of mimosine in *L. leucocephala*. Therefore, the stress conditions caused by the competition with the neighboring plant species may increase the production of mimosine, and the increased mimosine levels may also enhance the competitive ability of *L. leucocephala*.

Benzoic acid and cinnamic acid derivatives, such as *p*-hydroxybenzoic acid, gallic acid, vanillic acid, protocatechuic acid, *p*-hydroxyphenylacetic acid, *p*-hydroxycinnamic acid, caffeic acid, and ferulic acid, have been identified as allelochemicals in the leaves of *L. leucocephala* [183] (Figure 4). These compounds have also been identified as allelochemicals in various plant extracts and their rhizosphere soils [214,215,216]. They alter the structure and function of the plasma membranes, including their lipid and protein composition and transmembrane electrochemical potential. This results in membrane depolarization. These changes cause the non-specific influx and efflux of cations and anions, such as potassium, magnesium, phosphate, and nitrate ions, and affect the water balance of the plant cells [217,218]. These compounds also interfere with various metabolic processes, such as protein synthesis, photosynthesis, plant hormone synthesis, phytohormone synthesis, and various secondary metabolites, and inhibit cell division, resulting in reduced germination, growth, and development of the target plant species [214,215,216].

In addition, methanol extracts of *L. leucocephala* roots inhibited the nitrification process in soil, with gallocatechin being the most active component [219] (Figure 4). The first step in nitrification is the oxidation of ammonia (NH_3_) or ammonium (NH_4_) to nitrate (NO_2_**^−^**) by ammonia-oxidizing bacteria and archaea. The second step is the oxidation of nitrate to nitrite (NO_3_**^−^**) by nitrite-oxidizing bacteria, such as *Nitrobacter* spp. Nitrification is an important process for nitrogen cycling in ecosystems [220,221]. Gallocatechin from *L. leucocephala* may inhibit the nitrification process by inhibiting the associated bacterial activity, although the mechanism of action remains unknown. Inhibition of nitrification may reduce nitrate availability in the soil, resulting in the growth suppression of the neighboring plant species. Conversely, soil nitrogen levels under *L. leucocephala* stands were reported to be high due to its symbiotic nitrogen fixation [36,37,38]. Therefore, it will be necessary to evaluate the actual amount of nitrogen available in the soil under *L. leucocephala* stands in the future.

Based on these findings, *L. leucocephala* exhibits allelopathic activity and contains several allelochemicals, including mimosine, gallocatechin, and derivatives of benzoic and cinnamic acids. Mimosine accumulates in the medium and soil through the root exudation of *L. leucocephala*. Mimosine may also be released into the soil during the decomposition process of the plant residues. These allelochemicals described here can suppress the germination, growth, and fitness of neighboring competing plant species. As a result, *L. leucocephala* may obtain much more nutrients and water, increasing its growth and population.

*L. leucocephala* contains various other compounds, including terpenoids, benzenoids, flavonoids, saponins, tannins, steroids, and glycerides in the leaves, roots, stems and seeds [222,223,224,225,226,227,228,229,230,231,232]. Some of them have pharmacological effects, such as anti-tumor, anti-diabetic, anti-malaria, anti-inflammatory, anti-diarrhea, and antioxidant activities [225,227,230,231,232]. It is possible that some of these compounds are responsible for the unknown defense functions and contribute to the invasive properties of *L. leucocephala*.

## 7. Conclusions

*L. leucocephala* is listed in the world’s 100 worst alien invasive species, and has been spread to over 130 countries in the tropical, subtropical, and warm temperate regions. The species threatens native plant communities by reducing their density and richness in the infested areas. Its life history traits, such as year-round flowering and fruiting, self-fertility, prolific seed production, seed bank establishment, and adaptability to diverse environmental conditions may contribute to its infestation and population expansion.

Biotic stressors, such as herbivores, pathogens, and competing plant species, affect plant germination, growth, biomass, seed production, and senescence. These stressors exert significant selective pressures on plant survival, distribution, and abundance [233,234,235,236]. Defense mechanisms against these biotic stressors may be necessary for the invasive plant species to increase their distribution and abundance in new habitats [237,238,239]. *L. leucocephala* produces several toxic compounds that protect against herbivorous mammals and insects, parasitic nematodes, pathogenic fungi and bacteria, and neighboring competing plant species e.g., [81,82,143,151,183,216].

**Figure 6:**
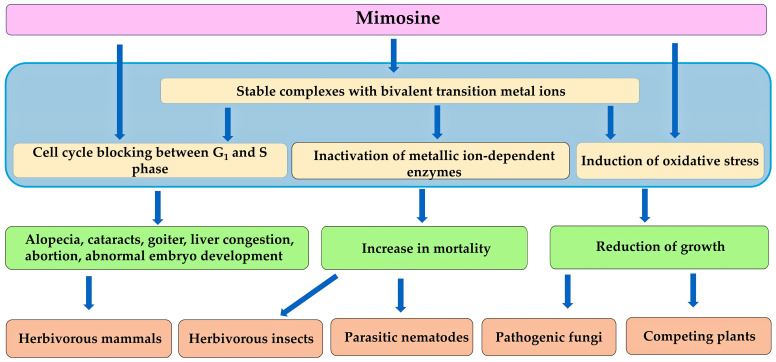
Mechanism of action of mimosine.

Mimosine is toxic to herbivorous mammals and insects, parasitic nematodes, pathogenic fungi, and competing plant species e.g., [79,124,132,142,150]. *L. leucocephala* contains high levels of mimosine in the entire plant [81,82,83]. Consumption of the plant parts and the administration of mimosine cause various pathological symptoms, including alopecia, cataracts, hepatic congestion, goiter, increases in the infertility, abortion, and abnormal embryo development in female mammals, and decrease in the serum testosterone levels in male mammals. These symptoms caused by mimosine may be due to the inactivation of several essential enzymes and the blockade of DNA replication [94,95,96,97,98,99,100,101,102,103,104,105,106,107,108,109,110,111,112,113]. Mimosine increases the mortality of insects and parasitic nematodes, and suppresses the growth of pathogenic fungi e.g., [123,124,132,150]. The activity of acetylcholinesterase and tyrosinase in insects was also suppressed by mimosine application [124]. In addition, mimosine acts as an allelochemical, inhibiting the growth of the competing plant species by blocking DNA replication and causing oxidative stress conditions [81,183] (Figure 6).

**Figure 7:**
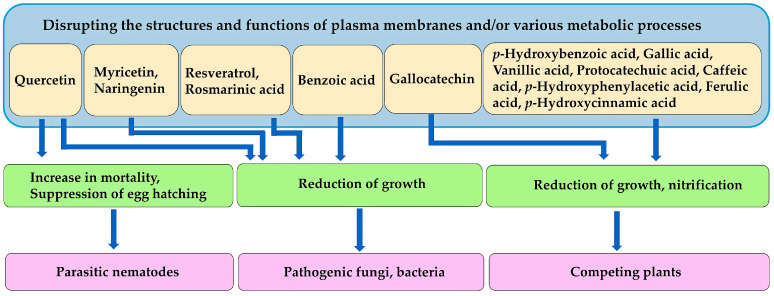
Mechanism of action of other compounds involved in the defense functions against parasitic nematodes, pathogenic fungi and bacteria, and competing plant species.

Quercetin exhibits nematocidal activity, inhibiting egg hatching, and increasing nematode mortality [143]. Benzoic acid, flavonoids, such as quercetin, naringenin, and myricetin, and polyphenolic compounds, such as rosmarinic acid and resveratrol, are toxic to pathogenic fungi and bacteria [151]. Several benzoic and cinnamic acid derivatives, including *p*-hydroxybenzoic acid, gallic acid, vanillic acid, protocatechuic acid, *p*-hydroxyphenylacetic acid, *p*-hydroxycinnamic acid, caffeic acid, and ferulic acid, act as allelochemicals, and inhibit the growth of neighboring competing plant species [183]. Gallocatechin suppresses the nitrification process by inhibiting related bacterial activity, reducing nitrate availability in the soil, and causing the growth suppression of neighboring plant species [216]. These compounds may cause the inhibition by disrupting the structures and functions of plasma membranes and/or various metabolic processes (Figure 7).

These compounds described herein may play a role in the defense functions against biotic stressors, such as herbivores, pathogens, and competing plant species. Since these biotic stressors exert significant selective pressure on plant survival, these compounds may represent the invasive traits of *L. leucocephala* that have undergone natural selection during the evolution of the species. They may contribute to the increased survival, growth, and abundance of *L. leucocephala* as an invasive plant species in its introduced ranges.
